# When Steroids Complicate the Diagnosis: Recurrent Steroid-Induced Psychosis, Severe Hyponatraemia With Generalized Tonic-Clonic Seizure, and Acute Lacunar Infarction During Treatment for Suspected Giant Cell Arteritis

**DOI:** 10.7759/cureus.113128

**Published:** 2026-07-21

**Authors:** Bola Reyad

**Affiliations:** 1 General Internal Medicine, St. Mary's Hospital, Isle of Wight NHS Trust, Newport, GBR

**Keywords:** central nervous system vasculitis, corticosteroid therapy, diagnostic challenge, generalized tonic-clonic seizure, giant cell arteritis, glucocorticoid toxicity, hyponatraemia, lacunar infarction, multidisciplinary management, steroid-induced psychosis

## Abstract

A 77-year-old woman with a history of palindromic rheumatism and a previous episode of steroid-induced psychosis was treated with high-dose prednisolone for suspected giant cell arteritis because of the risk of irreversible visual loss. Within days, she developed recurrent psychotic symptoms requiring hospital admission. During her admission, she experienced profound hypovolaemic hyponatraemia complicated by a generalized tonic-clonic seizure. Following correction of her sodium level, brain magnetic resonance imaging unexpectedly demonstrated an acute left basal ganglia lacunar infarction despite the absence of focal neurological deficits. The coexistence of recurrent steroid-induced psychosis, severe electrolyte disturbance, seizure, and acute cerebral infarction created a significant diagnostic challenge, prompting multidisciplinary involvement from rheumatology, neurology, stroke medicine, psychiatry, endocrinology, and general internal medicine. Further investigations, including CT angiography, cerebrospinal fluid analysis, autoimmune screening, and fluorodeoxyglucose positron emission tomography, were undertaken to evaluate for possible systemic or central nervous system vasculitis. This case highlights the importance of maintaining a broad differential diagnosis in patients receiving glucocorticoid therapy, particularly when neuropsychiatric symptoms coexist with metabolic and neurological abnormalities. It also illustrates the value of multidisciplinary assessment in distinguishing medication-related adverse effects from alternative or concurrent pathology.

## Introduction

Giant cell arteritis (GCA) is the most common large-vessel vasculitis in adults over 50 years of age and represents a medical emergency because delayed treatment may result in irreversible visual loss. Glucocorticoids remain the cornerstone of treatment, and current international guidelines recommend immediate corticosteroid therapy whenever GCA is strongly suspected, even before the diagnosis is confirmed [1]. Despite their therapeutic benefits, corticosteroids are associated with a broad spectrum of neuropsychiatric adverse effects, ranging from mild mood disturbance and insomnia to severe psychosis, delirium and cognitive impairment [2,3]. Although steroid-induced psychosis is a well-recognised complication of glucocorticoid therapy, recurrent episodes following re-exposure may present a considerable diagnostic challenge, particularly when accompanied by concurrent metabolic or neurological abnormalities. The risk increases with higher corticosteroid doses, and a previous episode of steroid-induced psychosis is recognised as an important risk factor for recurrence.

Hyponatraemia is another important cause of acute neuropsychiatric deterioration in older adults and, when severe, may lead to seizures, encephalopathy and altered mental status [4]. Among the recognised causes of hyponatraemia, syndrome of inappropriate antidiuretic hormone secretion (SIADH) is a common mechanism in hospitalised patients and may result in progressive cerebral oedema with neurological manifestations when severe. The coexistence of steroid-induced psychiatric symptoms, profound electrolyte disturbance and acute cerebral infarction can obscure the underlying diagnosis and complicate clinical decision-making. Furthermore, in patients with suspected GCA who develop new neurological manifestations, primary central nervous system vasculitis should be considered as an important differential diagnosis, although it remains an uncommon condition that requires careful exclusion of more frequent mimics through appropriate clinical, laboratory and radiological evaluation [5].

We present the case of a 77-year-old woman with a previous history of steroid-induced psychosis who developed recurrent neuropsychiatric symptoms shortly after commencing high-dose prednisolone for suspected GCA. Her admission was further complicated by severe hyponatraemia, a generalized tonic-clonic seizure and the unexpected finding of an acute lacunar infarction on brain magnetic resonance imaging. Extensive multidisciplinary assessment was undertaken to exclude cerebral vasculitis before her presentation was considered most consistent with recurrent steroid-induced psychosis complicated by severe hyponatraemia and an acute lacunar infarction of uncertain relationship to the acute presentation. This case highlights the importance of maintaining a broad differential diagnosis in patients receiving glucocorticoid therapy and demonstrates the value of close collaboration between internal medicine, psychiatry, neurology and rheumatology when multiple potentially interacting pathological processes coexist.

## Case presentation

A 77-year-old woman with a medical history of type 2 diabetes mellitus, hypertension, atrial fibrillation anticoagulated with apixaban, previous transient ischaemic attack, asthma, diabetic retinopathy, and palindromic rheumatism under rheumatology follow-up presented with a new-onset unilateral temporal headache. Owing to the clinical suspicion of GCA and the risk of irreversible visual loss, she was commenced on oral prednisolone 40 mg once daily despite a documented history of prednisolone-induced psychosis during previous corticosteroid treatment. The prednisolone dose was subsequently tapered to 20 mg and later to 10 mg because of worsening neuropsychiatric symptoms. The patient's corticosteroid dose adjustments and other medications relevant to the diagnostic assessment and clinical management are summarised in Table 1, and the chronological sequence of the patient's presentation, investigations, multidisciplinary assessment, and management is summarised in Table 2.

**Table 1:** Corticosteroid Dose Adjustments and Relevant Medications During Hospital Admission This table summarises the principal medications relevant to the patient's presentation and management during admission. It highlights corticosteroid dose adjustments following the development of neuropsychiatric symptoms together with other medications that influenced the diagnostic assessment or ongoing clinical management. It is not intended to represent a complete medication reconciliation.

Medication	Indication	Dose	Clinical relevance
Prednisolone	Suspected giant cell arteritis (GCA)	40 mg once daily	Initiated because of suspected GCA despite previous steroid-induced psychosis.
Prednisolone	Dose reduction	20 mg once daily	Reduced following worsening neuropsychiatric symptoms.
Prednisolone	Dose reduction	10 mg once daily	Further reduced during admission.
Lorazepam	Agitation and insomnia	As required	Commenced following liaison psychiatry review.
Risperidone	Psychotic symptoms	Low dose if required	Reserved if psychotic symptoms progressed.
Apixaban	Atrial fibrillation	Long-term treatment	Continued throughout admission.

**Table 2:** Timeline of Clinical Events This timeline summarises the chronological sequence of the patient's presentation, investigations, multidisciplinary assessment and clinical management throughout the admission.

Time	Clinical event
Initial presentation	New unilateral temporal headache with clinical suspicion of giant cell arteritis (GCA).
Day 0	Prednisolone 40 mg once daily commenced.
Following corticosteroid initiation	Progressive confusion, insomnia, pressured speech, erratic behaviour and disorganised thought developed.
Hospital admission	Recurrent steroid-induced psychosis suspected. Severe hypertension and hyponatraemia (serum sodium 116 mmol/L) identified.
During admission	Serum sodium declined to a nadir of 104 mmol/L with associated hypokalaemia and hypophosphataemia.
During admission	Generalized tonic-clonic seizure occurred.
After stabilisation	Brain MRI demonstrated an acute left basal ganglia lacunar infarction.
Further investigations	CT angiography, cerebrospinal fluid analysis and autoimmune investigations showed no convincing evidence of cerebral vasculitis.
Multidisciplinary review	Recurrent corticosteroid-induced psychosis remained the most likely diagnosis; GCA remained a clinical diagnosis while further outpatient investigations were arranged.
Discharge	Clinical improvement following electrolyte correction and corticosteroid dose reduction. Follow-up arranged with rheumatology, neurology, psychiatry and stroke services.

She was admitted several days after commencing corticosteroid therapy with progressive confusion, erratic behaviour, pressured speech, insomnia, and increasingly disorganised thought processes. Her husband reported escalating preoccupation with her physical health and increasing concern that healthcare professionals were underestimating the severity of her symptoms. On admission, recurrent corticosteroid-induced psychosis was considered the leading diagnosis because of the close temporal relationship between symptom onset and corticosteroid exposure together with her previous history. Liaison psychiatry was involved early during admission, and lorazepam was commenced for agitation and insomnia, while low-dose risperidone was reserved in the event of progression of psychotic symptoms.

Initial examination demonstrated marked hypertension (224/130 mmHg) but no focal neurological deficit or visual impairment. Given the marked hypertension and acute encephalopathy, hypertensive emergency with posterior reversible encephalopathy syndrome (PRES) was also considered. However, subsequent brain magnetic resonance imaging (MRI) demonstrated an acute lacunar infarction without imaging features to suggest PRES. There was no temporal artery tenderness, although the left temporal artery appeared mildly prominent. Cranial nerve examination, motor function, sensation, coordination, and gait were unremarkable. Non-contrast computed tomography of the head demonstrated no acute intracranial abnormality. Initial blood tests demonstrated severe hyponatraemia (serum sodium 116 mmol/L; reference range 135-145 mmol/L), and she was managed with close biochemical monitoring and supportive medical treatment.

Despite treatment, her electrolyte disturbance progressively worsened during admission. Serum sodium continued to decline to a nadir of 104 mmol/L (reference range 135-145 mmol/L), accompanied by hypokalaemia (2.6 mmol/L; reference range 3.5-5.3 mmol/L) and hypophosphataemia (0.55 mmol/L; reference range 0.80-1.50 mmol/L). Following clinical assessment, SIADH was considered the most likely explanation for the persistent hyponatraemia. During this period, she developed a generalized tonic-clonic seizure associated with transient impairment of consciousness. Owing to the severity of the electrolyte abnormalities, she was reviewed by the intensive care team for possible admission and central venous access to facilitate electrolyte replacement. Although a right subclavian central venous catheter was inserted successfully, intensive care admission was not considered necessary because she remained haemodynamically stable, and electrolyte replacement was considered achievable safely on the ward.

**Figure 1:**
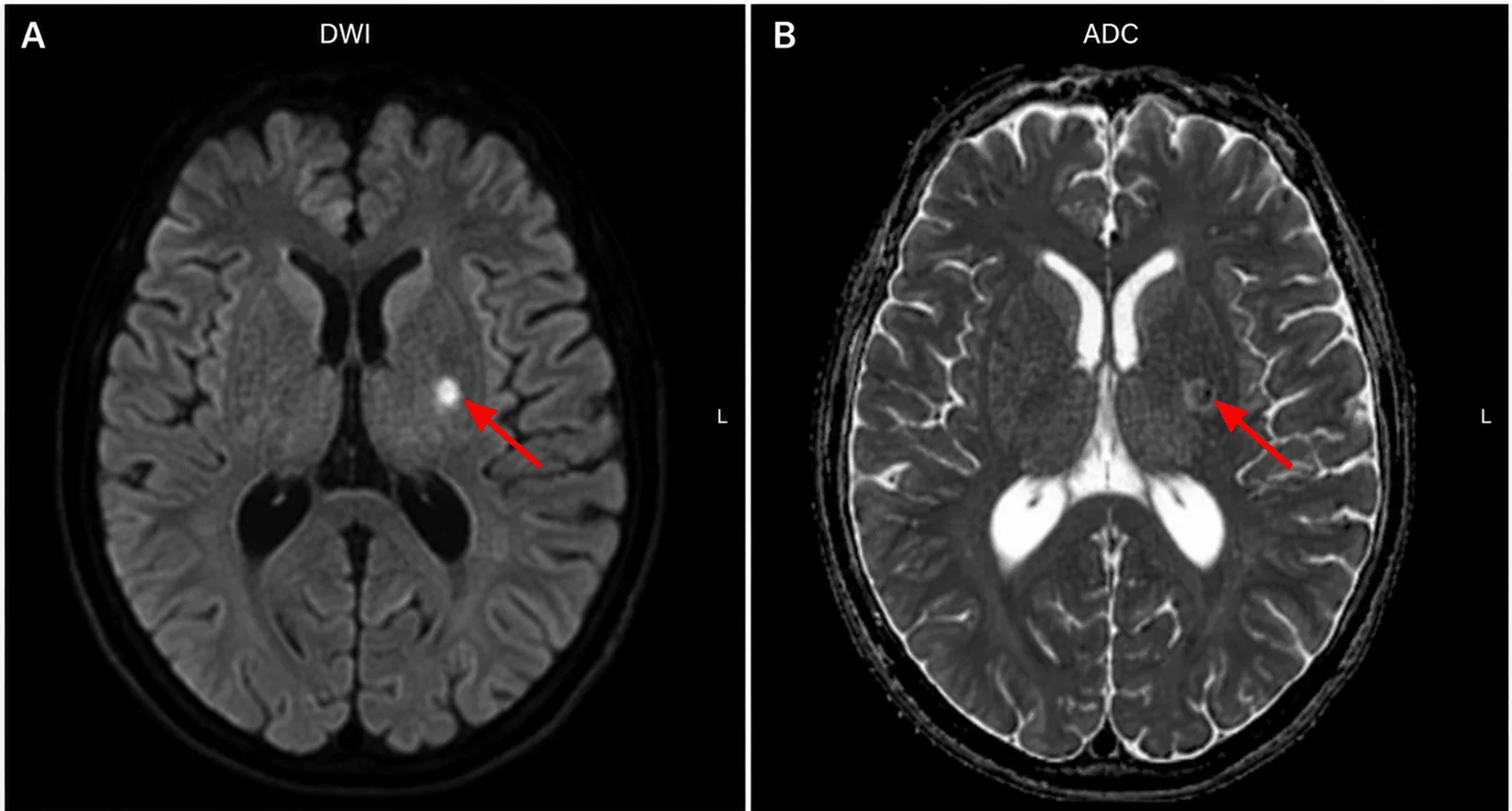
Brain MRI Demonstrating an Acute Left Basal Ganglia Lacunar Infarction Axial brain MRI demonstrating an acute lacunar infarction in the left basal ganglia. (A) Diffusion-weighted imaging (DWI) shows a focal area of restricted diffusion in the left basal ganglia/internal capsule region (red arrow). (B) The corresponding apparent diffusion coefficient (ADC) map demonstrates low signal intensity in the same location (red arrow), confirming true restricted diffusion consistent with an acute lacunar infarction.

Following correction of the electrolyte disturbance and neurological stabilisation, MRI of the brain was performed to investigate persistent neuropsychiatric symptoms and exclude an underlying structural cause for her seizure. The examination unexpectedly demonstrated a small acute lacunar infarction involving the left basal ganglia adjacent to the posterior limb of the internal capsule (Figure 1). Despite this radiological finding, repeated neurological examinations demonstrated no persistent facial weakness, limb weakness, sensory loss, dysarthria, ataxia, or gait disturbance attributable to the infarction.

The identification of an acute cerebral infarction in a patient with suspected GCA, recurrent corticosteroid-induced psychosis, severe hyponatraemia, and seizure substantially broadened the differential diagnosis. An extensive multidisciplinary assessment involving rheumatology, neurology, stroke medicine, psychiatry, endocrinology, and general internal medicine was undertaken to evaluate for inflammatory, vascular, metabolic, and medication-related causes of her presentation. CT angiography of the intracranial and cervical vessels demonstrated no radiological evidence of vasculitis or significant arterial stenosis. Lumbar puncture performed under fluoroscopic guidance demonstrated cerebrospinal fluid protein of 0.37 g/L (reference range 0.15-0.45 g/L), glucose of 6.8 mmol/L (reference range approximately 2.5-4.4 mmol/L or >60% of simultaneous serum glucose), lactate of 2.7 mmol/L (reference range 1.1-2.4 mmol/L), and negative xanthochromia, with no evidence of central nervous system infection or inflammatory pathology. Autoimmune investigations, including antinuclear antibody, antineutrophil cytoplasmic antibody, and anti-cyclic citrullinated peptide antibody testing, were negative, while HIV and hepatitis screening were unremarkable. Neurology review concluded that there was minimal evidence to support a diagnosis of cerebral vasculitis. Fluorodeoxyglucose positron emission tomography and temporal artery ultrasound were requested to evaluate for large-vessel involvement and to further investigate the clinical suspicion of GCA.

Following multidisciplinary assessment and ongoing medical management, electrolyte abnormalities gradually improved, with serum sodium increasing from a nadir of 104 mmol/L to 127 mmol/L (reference range 135-145 mmol/L). Corticosteroid therapy was reviewed jointly with the rheumatology team, and her neuropsychiatric symptoms improved with supportive medical and psychiatric management. No further seizures occurred during the remainder of her admission. The diagnosis of GCA remained a clinical diagnosis throughout admission while further outpatient investigations were arranged. Following multidisciplinary review, recurrent corticosteroid-induced psychosis remained the most likely explanation for her neuropsychiatric presentation, while profound hyponatraemia and the acute lacunar infarction, of uncertain relationship to the acute presentation, were recognised as important concurrent pathologies that complicated the diagnostic process. She was discharged with coordinated follow-up involving rheumatology, neurology, psychiatry, and stroke services.

## Discussion

The present case illustrates the diagnostic complexity that may arise when multiple neurological and medical complications occur simultaneously following corticosteroid therapy. Current international guidelines recommend immediate initiation of high-dose glucocorticoids when GCA is suspected to minimise the risk of irreversible visual loss, even before the diagnosis is confirmed [1,6]. While this approach is clinically appropriate, clinicians must remain vigilant for significant treatment-related adverse effects.

Steroid-induced psychosis is a well-recognised complication of glucocorticoid therapy and typically develops within days to weeks of treatment initiation, particularly following exposure to higher doses [2,3]. Clinical manifestations range from mood changes and insomnia to mania, delirium and frank psychosis. Recurrent episodes following re-exposure, as demonstrated in our patient, have been described but remain relatively uncommon. The temporal relationship between corticosteroid initiation and symptom recurrence, together with a documented previous episode of steroid-induced psychosis, strongly supported glucocorticoid therapy as the principal precipitating factor. Alternative causes of her neuropsychiatric presentation were considered throughout admission but became less likely following multidisciplinary assessment and clinical improvement after corticosteroid dose reduction and correction of the metabolic disturbance.

The patient's presentation was further complicated by profound hyponatraemia resulting in a generalized tonic-clonic seizure. Severe hyponatraemia is a neurological emergency and may present with confusion, behavioural disturbance, seizures and impaired consciousness, frequently mimicking or exacerbating primary psychiatric illness [4]. In our patient, SIADH was considered the most likely explanation for the persistent hyponatraemia, although electrolyte disturbances in acutely unwell older adults are often multifactorial. Consequently, metabolic abnormalities should always be actively excluded in patients presenting with acute psychiatric symptoms, particularly when neurological deterioration occurs during admission.

The unexpected MRI finding of an acute lacunar infarction considerably broadened the differential diagnosis. In the context of suspected GCA, this prompted concern regarding possible cerebral vasculitis. Primary central nervous system vasculitis remains an uncommon disorder with heterogeneous clinical and radiological manifestations, and its diagnosis requires careful correlation of clinical findings with cerebrospinal fluid analysis, vascular imaging and exclusion of alternative causes [5]. In our patient, normal CT angiography, unremarkable cerebrospinal fluid studies and subsequent clinical improvement following correction of electrolyte abnormalities and corticosteroid dose reduction argued against an inflammatory cerebral vasculitic process. Although the acute lacunar infarction required careful evaluation, the absence of persistent focal neurological deficits together with the lack of radiological and cerebrospinal fluid evidence of cerebral vasculitis suggested that it was unlikely to account for the overall clinical presentation. Although GCA remained the working clinical diagnosis, there was little evidence to support cerebral vasculitis as the cause of the patient's neurological deterioration.

This case highlights the importance of avoiding diagnostic anchoring when patients receiving corticosteroid therapy develop acute neuropsychiatric deterioration. Steroid-induced psychosis, severe electrolyte disturbance and structural neurological abnormalities may coexist, creating substantial diagnostic uncertainty. Sequential clinical reassessment and close multidisciplinary collaboration between internal medicine, psychiatry, neurology and rheumatology were essential in establishing the most likely diagnosis and guiding appropriate management while avoiding unnecessary escalation of immunosuppressive therapy. As a single case report, definitive causal relationships cannot be established, and the relative contribution of each pathological process to the patient's presentation cannot be determined with certainty.

## Conclusions

This case demonstrates the diagnostic complexity that can arise when recurrent corticosteroid-induced psychosis is accompanied by severe metabolic and neurological complications. Although the patient's neuropsychiatric symptoms were initially attributed to corticosteroid therapy, profound hyponatraemia, a generalized tonic-clonic seizure, and the unexpected finding of an acute lacunar infarction necessitated repeated reassessment of the working diagnosis and a comprehensive multidisciplinary investigation to exclude inflammatory cerebrovascular disease. The absence of supportive clinical, radiological, and cerebrospinal fluid findings for cerebral vasculitis emphasised the importance of avoiding premature diagnostic closure and maintaining a broad differential diagnosis in patients with suspected GCA who deteriorate following corticosteroid therapy. Careful clinical reassessment, timely neuroimaging, correction of metabolic abnormalities, and close collaboration between multiple specialties were essential in establishing the most likely diagnosis and guiding appropriate management. As a single case report, this case cannot establish definitive causal relationships but highlights the importance of systematic reassessment when multiple concurrent pathologies complicate the clinical picture.
